# Distribution of Pd clusters on ultrathin, epitaxial TiO*_x_* films on Pt_3_Ti(111)

**DOI:** 10.3762/bjnano.6.204

**Published:** 2015-10-09

**Authors:** Christian Breinlich, Maria Buchholz, Marco Moors, Tobias Pertram, Conrad Becker, Klaus Wandelt

**Affiliations:** 1 Institute of Physical and Theoretical Chemistry, University of Bonn, Wegelerstraße 12, D-53115 Bonn, Germany; 2 Institute of Functional Interfaces, Karlsruhe Institute of Technology, D-76021 Karlsruhe, Germany; 3 Peter Grünberg Institute, Forschungszentrum Jülich, Wilhelm-Johnen-Straße, D-52425 Jülich, Germany; 4 Aix Marseille Université, CNRS, CINaM UMR 7325, 13288 Marseille, France; 5 Institute of Experimental Physics, University of Wroclaw, Pl. Maksa Borna, 50-204 Wroclaw, Poland

**Keywords:** cluster growth, palladium, platinum–titanium alloy, scanning tunnelling microscopy (STM), template, titanium oxide

## Abstract

Scanning tunnelling microscopy (STM) was used to investigate the nucleation and growth of palladium clusters on two different, ultrathin, epitaxial, titania films grown on a Pt_3_Ti(111) surface. The first oxide phase, z'-TiO*_x_*, is anisotropic and consists of parallel stripes separated by trenches. Defects (i.e., oxygen vacancies) in this structure are confined to these trenches and act as nucleation sites. Therefore, the Pd clusters are mostly arranged in unidirectional rows along the trenches, creating a template effect. The second phase, w'-TiO*_x_*, exhibits a hexagonal, long range, (7 × 7)R21.8°, Moiré-type superstructure with fewer and shallower defects, making the template effect less discernible.

## Introduction

Catalysts often consist of metal nanoparticles dispersed on an oxide support structure. Small metal particles on an insulating substrate exhibit different electronic properties than the corresponding bulk metal phase. Moreover, in many cases, the oxide surface not only acts as a support structure but also takes part in the reaction cycle, for example, in oxygen spillover to the metal particles [1–2]. Thus, the investigation of the properties of supported metal clusters and the influence of the metal-oxide interfaces are of great interest. In particular, titanium oxides are often correlated with the so-called strong metal support interaction (SMSI) effect, which describes the influence of a transition metal oxide support on noble metal clusters yielding novel catalytic properties [1–7]. In order to understand this effect, well-defined model systems are needed. “Well-defined” refers to both the clusters and the supporting substrate, as exemplified in [8–10] for example. In the ideal case, the clusters should be monodisperse or have at least a known size distribution. Ideal oxide surfaces can be favourably implemented in the form of thin epitaxial films. Such films grown on a single crystalline, metal support have several advantages: (a) standard surface science techniques can be applied due to the high conductivity of these films compared to the respective bulk oxides, (b) the films can be prepared with a very high degree of structural preciseness, and (c) the influence of bulk effects such as subsurface oxygen vacancies is excluded.

In this sense we concentrate here on the preparation of uniform Pd clusters on two different, ultrathin, epitaxial TiO*_x_* films grown on a chemically ordered, Pt_3_Ti(111), single crystal surface. One of the TiO*_x_* films has a rectangular structure and the other a hexagonal structure. In a recent publication we described the detailed protocol on how to grow these TiO*_x_* films by direct oxidation of the Pt_3_Ti(111) surface at elevated temperatures [6]. Granozzi et al., who found very similar phases by “reactive evaporation” of titanium onto a Pt(111) surface in oxygen [7], introduced the notation z'-TiO*_x_* (zigzag-like) for the rectangular and w'-TiO*_x_* (wagon-wheel-like) for the hexagonal oxide phase, according to their appearance in STM images. Due to the similarity of our films to those described for Pt(111), we decided to simply adopt the same nomenclature throughout this paper. The rectangular z'-TiO*_x_* phase obtained on the Pt_3_Ti(111) surface exhibits characteristic stripes of densely packed, Ti–O rows of bilayer thickness. These are separated 1.44 nm apart from each other with parallel trenches in between. The w'-TiO*_x_* phase consists of a hexagonal, oxygen-terminated, Ti–O bilayer, which shows similarities to the Moiré superstructure of ultrathin aluminium oxide films grown on the chemically ordered, Ni_3_Al(111) surface [11–14]. The advantages of growing an oxide film from a component of an ordered alloy surface with a higher enthalpy of oxide formation are the somewhat better structural quality of the resulting films and their improved reproducibility compared to films grown by “reactive evaporation” [7].

Both the z'-TiO*_x_* phase on Pt(111) and the Moiré superstructure of the alumina film on Ni_3_Al(111) have already been proven to be excellent templates for the ordered growth of metal clusters. Granozzi et al. found ordered rows of Fe and Au clusters along the trenches of the z'-TiO*_x_* phase [15–16], while other studies (also of our own group) demonstrated the growth of ordered arrays of, for example, Pd-, Au-, and Fe- [11–14] or Co-clusters on Al_2_O_3_/Ni_3_Al(111) [10]. In the present paper we investigate the template effect of two different structures of the same type of oxide on the cluster growth of the same metal, namely Pd on z'-TiO*_x_* and w'-TiO*_x_*.

## Experimental setup

The scanning tunnelling microscopy (STM) experiments were conducted on our custom-built LT-STM, which for the experiments presented in this paper, was run at room temperature. The sample was prepared in an adjacent preparation chamber, which was equipped with a sputter gun, low energy electron diffraction (LEED) optics and an Auger electron spectroscopy (AES) analyser. The STM tips were electrochemically etched from a 0.5 mm tungsten wire and cleaned under ultra-high vacuum (UHV) conditions using voltage pulses of 10 ms duration between −10 and +10 V. The STM data were analysed with the WSxM freeware program [17].

The (111)-oriented Pt_3_Ti crystal was purchased from MaTeck (Jülich, Germany) and cleaned using several cycles of argon sputtering at 900 K for 10 min with subsequent annealing at 1100 K for another 10 min in order to restore the surface structure. The crystallographic and chemical order of the surface was verified by a sharp (2 × 2) superstructure visible in LEED experiments and the absence of any carbon or oxygen contamination in AES experiments.

Palladium was evaporated from a simple Knudsen cell with a slow deposition rate of approximately 2 · 10^−3^ MLs^−1^. The amount deposited was controlled by the deposition time and monitored by the evolution of the 330 eV Pd AES signal in relation to the 505 eV oxygen signal, as well as by the number density and size of the metal clusters seen in the STM images. The surface was kept at room temperature during evaporation and no post-deposition annealing was carried out.

## Results and Discussion

### The rectangular z'-TiO*_x_* (zig-zag-like) phase

**Figure 1:**
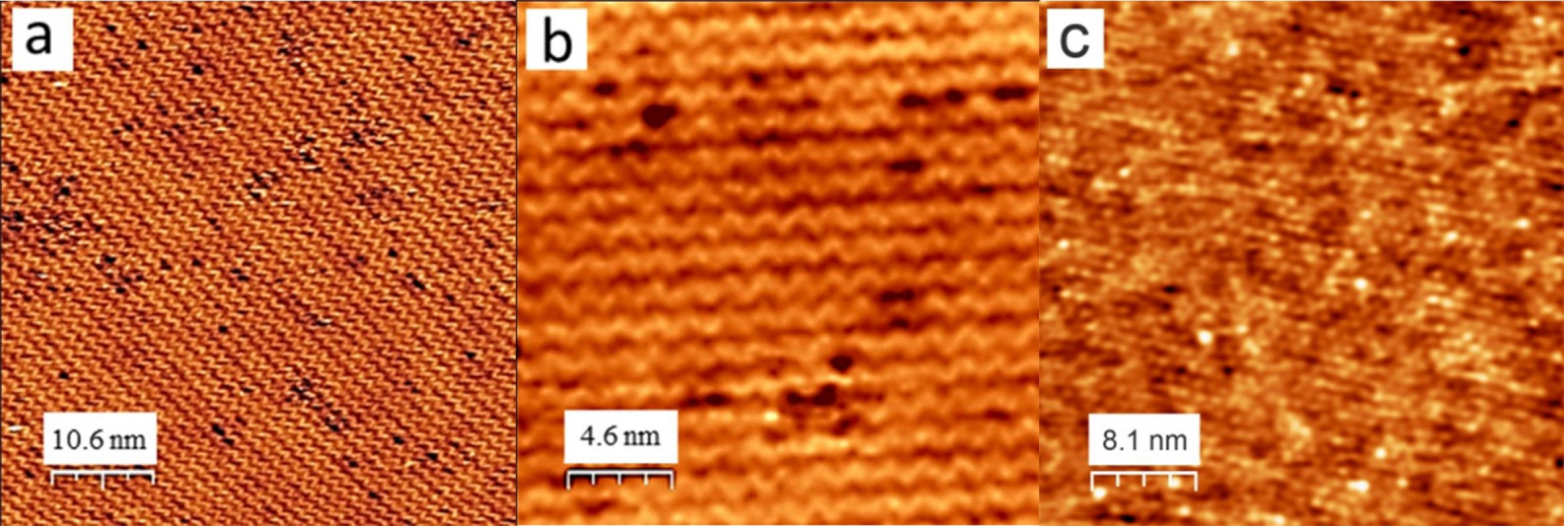
STM image of the z'-TiO*_x_* phase: (a) 53.3 × 53.3 nm^2^, bias voltage *U*_B_ = 0.91 V, *I*_T_ = 50 pA, (b) 22.8 × 22.8 nm^2^, *U*_B_ = 0.99 V, *I*_T_ = 40 pA, and (c) STM image of the w'-TiO*_x_* phase (57.1 × 57.1 nm^2^, *U*_B_ = −1.08 V, *I*_T_ = 55 pA). The images in (a) and (c) have been adapted with permission from [6], copyright 2014 American Chemical Society.

Exposing the clean Pt_3_Ti(111) surface to rather small amounts of oxygen gas (less than 200 L dosed in the 10^−8^ mbar range) at temperatures between 800 and 1100 K results in the formation of the z'-TiO*_x_* phase [6]. This atomically thin, titanium oxide phase is characterized by a typical striped pattern in the STM images. Former LEED measurements have shown a commensurate rectangular unit cell with a (6 × 3√3) superstructure with respect to the (1 × 1) spots of the alloy surface and a unit cell size of (16.6 ± 0.2) × (14.4 ± 0.2) Å, while high resolution electron energy loss spectroscopy (HREELS) and X-ray photoelectron spectroscopy (XPS) measurements indicated the existence of an oxygen-terminated Ti–O bilayer [6–7]. Depending on the applied oxygen dose, the z'-TiO*_x_* phase covers different fractions of the crystal surface, ranging from small separated islands up to a nearly complete film covering the whole surface. Figure 1a,b shows STM images of such an ultrathin oxide film, which was prepared by dosing 45 L of oxygen while holding the sample temperature at 1000 K. This amount of oxygen was sufficient to cover more than 80% of the alloy surface with z'-TiO*_x_*. The stripes, which are approximately 1.4 nm apart from each other and separated by darker trenches, show the characteristic zigzag motif after which this structure was named. Inside the bright stripes the density of titanium atoms is higher than in the trenches, which is a result of the lattice mismatch between the titanium oxide film and the metallic substrate.

As suggested by theoretical calculations, the stripes consist of titanium atoms of different coordination [18]. Using a negative tip potential, four-fold oxygen-coordinated Ti atoms appear with the highest contrast in the STM images. They are surrounded by less bright, three-fold oxygen-coordinated Ti atoms. This mixture of Ti atoms of different electronic density is responsible for the characteristic striped pattern of the z'-TiO*_x_* phase [18]. The STM images in Figure 1a,b also show several defects with the appearance of small black holes and a rough depth of 40 pm, which are all located within the trenches. At these point defects, titanium and oxygen atoms are probably missing and, thus, the bare metallic substrate is exposed [7]. Growth experiments with Au and Fe have already shown that both the trenches and, in particular, these defect sites within the trenches are preferred nucleation sites for cluster growth [15–16].

### Pd cluster growth on the z'-TiO*_x_* phase

**Figure 2:**
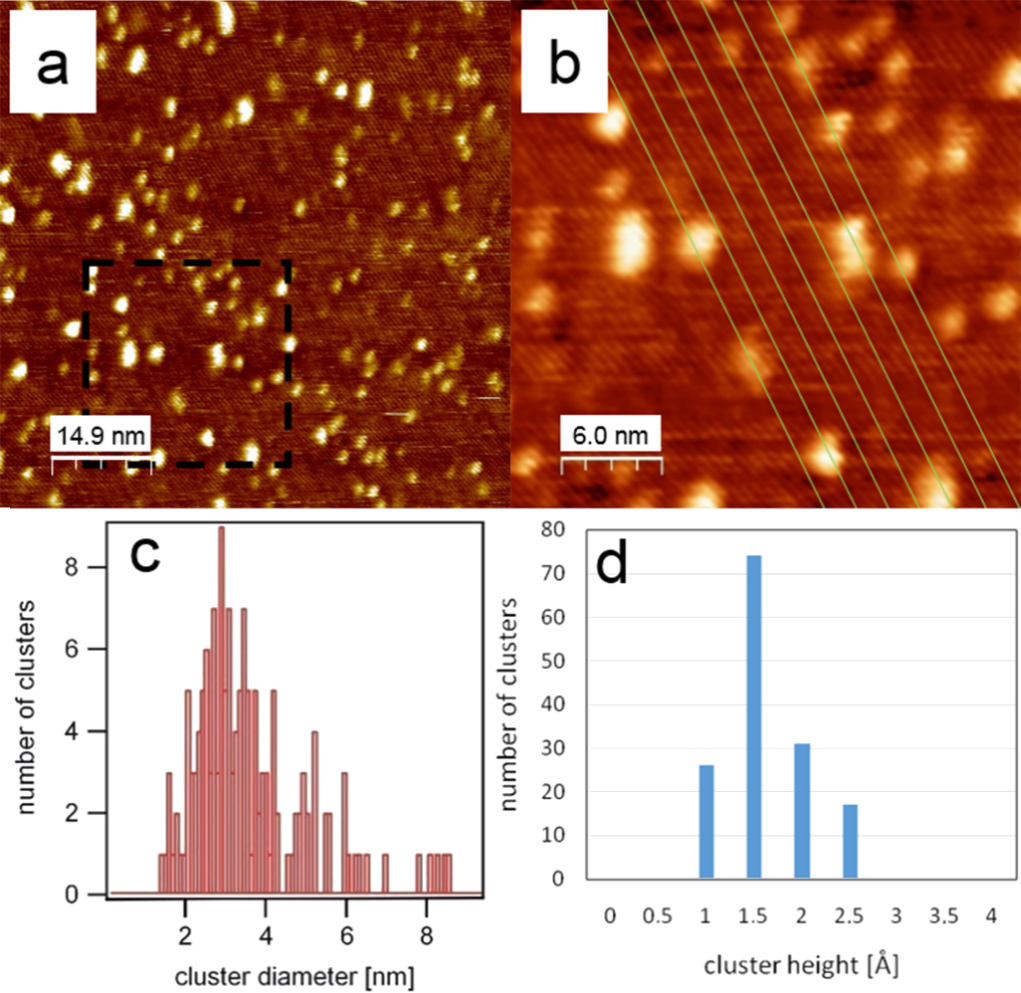
(a) STM image of Pd clusters grown at room temperature on a z'-TiO*_x_* covered surface with a Pd coverage of 0.1 ± 0.05 ML after 90 s of Pd deposition (76.1 × 76.1 nm^2^, *U*_B_ = 0.8 V, *I*_T_ = 56 pA), (b) detailed view for the marked section from image (a), (c) corresponding size distribution and (d) height distribution of Pd clusters. The green lines in (b) mark the trenches of the z'-TiO*_x_* substrate.

Figure 2a shows an STM image of a z'-TiO*_x_* surface onto which Pd was deposited at room temperature for 90 s (≈0.1 ± 0.05 ML). The stripes and trenches of the z'-TiO*_x_* structure are visible in Figure 2a running from the upper left to the lower right of the image. In the close-up Figure 2b, the direction and separation of the trenches are accentuated by parallel lines. The individual Pd clusters are nearly all located within the trenches between the stripes. Only a few larger elongated clusters are visible. The tilted orientation of their long axis with respect to the trench orientation may be suggestive of two coalesced clusters at adjacent defects in two neighbouring stripes. They are not equally spaced within the trenches and appear more or less randomly distributed along the trenches. Most of the clusters are imaged larger than the width of the trenches, but their centres are always within the trenches. The distribution of the cluster diameters and cluster heights as taken from Figure 2a are displayed in Figure 2c,d and show a mean diameter of 3.5 ± 1.6 nm and a height of 1.5–2 Å. It should be noted, however, that the measured particle diameter is actually the result of a convolution between the true particle size and the STM tip shape [14]. The actual particle diameter is smaller, but the distribution obtained for different coverages and the two different substrates still allow for comparison. Regarding the measured heights of 1.5–2 Å, it must be considered that this may not be the true geometric height due to electronic effects. However, it is safe to assume that the Pd particles are only one layer thick. These results are in good agreement with the published results of Au clusters grown on the corresponding z'-TiO*_x_* phase on Pt(111) [15].

The observed arrangement of the clusters is a consequence of the diffusion behaviour of the deposited metal atoms on this structurally anisotropic oxide surface. On the one hand the palladium–oxygen interaction is not very strong, permitting metal atom mobility at room temperature [19]. On the other hand, the trenches (1D depressions) guide the Pd atom diffusion preferentially along the grooves. Such anisotropic diffusion behaviour has been observed in the inverse system, namely the oxidation of Pd(110) leading to the growth of pronounced, elongated, oxide islands [20]. Thus, in the initial phase of the cluster growth, two competing processes take place: The defects, which are distributed randomly within (and only within) the trenches, will trap some Pd atoms and thereby act as heterogeneous nucleation sites. In principle, homogeneous nucleation may also occur at other places within the trenches when two or three diffusing Pd atoms meet and form an immobile nucleus. However, in the case of low coverage as studied here, heterogeneous nucleation dominates. Counting the number of visible defects in typical images of the z'-TiO*_x_* phase such as in Figure 1a leads to a typical defect density of approximately 3.9·10^4^ defects per µm^2^. Comparing this value with a typical cluster density of 3.7∙10^4^ clusters per µm^2^ (calculated by counting the Pd clusters in Figure 2a) supports our observation that the cluster formation on the surface is directly related to the defects along the trenches of the z'-TiO*_x_* phase.

The result is an anisotropic template effect of the z'-TiO*_x_* phase: The clusters are arranged in lines along the trenches, but not equally spaced within the trenches or across the stripes in neighbouring trenches.

### The hexagonal w'-TiO*_x_* (wagon-wheel-like) phase

The w'-TiO*_x_* phase is the second stable titanium oxide phase that can be grown on the Pt_3_Ti(111) surface with oxygen doses higher than 200 L within a temperature window of 800–1100 K [6]. HREELS and XPS studies also indicated a Ti–O bilayer structure with oxygen termination as a basic structure element for this phase [6–7]. LEED measurements revealed a hexagonal, higher-order commensurate (7 × 7)R21.8° superstructure with unit cell lattice vectors of 19.4 ± 0.2 Å. In contrast to the z'-TiO*_x_* phase, however, it always covers the entire surface. This can be seen as the crucial factor for the symmetry change. At complete surface coverage, it is energetically more favourable for the oxide film to adopt the hexagonal symmetry of the substrate. This is normally not very common for titanium oxides and makes the w'-TiO*_x_* phase a very interesting object of investigation. For the experiments presented here, the oxide film was prepared by exposing the sample to a temperature of 1000 K with 400 L of oxygen at a pressure of 1∙10^−7^ mbar. The STM image in Figure 1c shows a hexagonal arrangement of bright protrusions with nearly the same lattice parameters as measured by LEED [6]. This structure can be interpreted as a Moiré pattern that arises from the coincidence of the lattices of the metal support and the oxide overlayer. A Moiré pattern with a (7 × 7)R21.8° superstructure can be created by superimposing a hexagonal oxide layer with a unit cell size of 3.18 Å on the metal substrate with a unit cell size of 2.76 Å, rotated by 3.5° [7]. Those positions where the oxygen atoms are situated on top or on bridge sites of the metal substrate lattice are imaged brighter in the STM when using a negative tip potential. The number density of defects that exhibit an apparent depth of 18 pm is significantly lower than on the z'-TiO*_x_* surface, as visualized later in Figure 5b,d.

#### Pd cluster growth on the w'-TiO*_x_* phase

**Figure 3:**
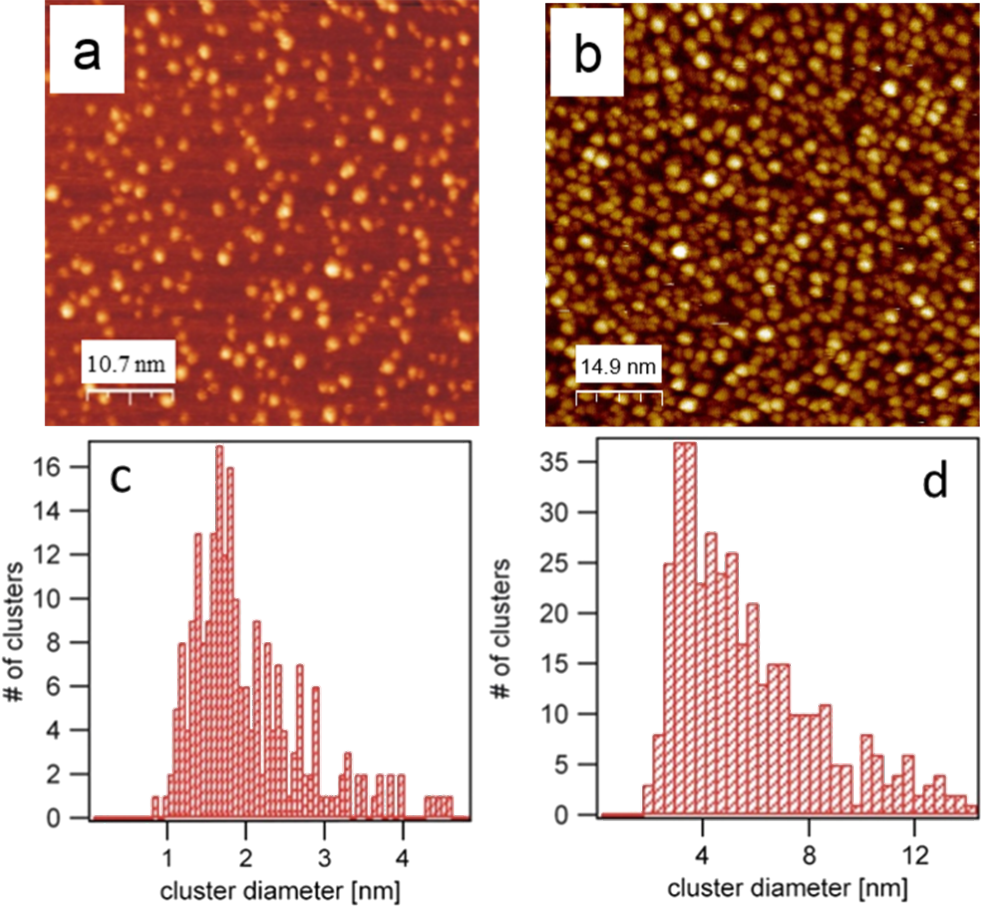
Pd clusters grown at RT on the w'-TiO*_x_* phase: (a) low Pd coverage of 0.15 ± 0.02 ML after 90 s of Pd deposition (53.3 × 53.3 nm^2^, *U*_B_ = 0.9 V, *I*_T_ = 53 pA), (b) high Pd coverage of 0.73 ± 0.05 ML after 420 s of Pd deposition (76.1 × 76.1 nm^2^, *U*_B_ = 2.13 V, *I*_T_ = 57 pA, (c) size distribution of the cluster from (a), and (d) size distribution of the clusters from (b).

Figure 3 shows two STM images obtained at comparatively low (≈0.15 ML, Figure 3a) and high (≈0.75 ML, Figure 3b) Pd coverages on the w'-TiO*_x_* surface obtained after 90 s and 420 s of deposition, respectively.

**Figure 4:**
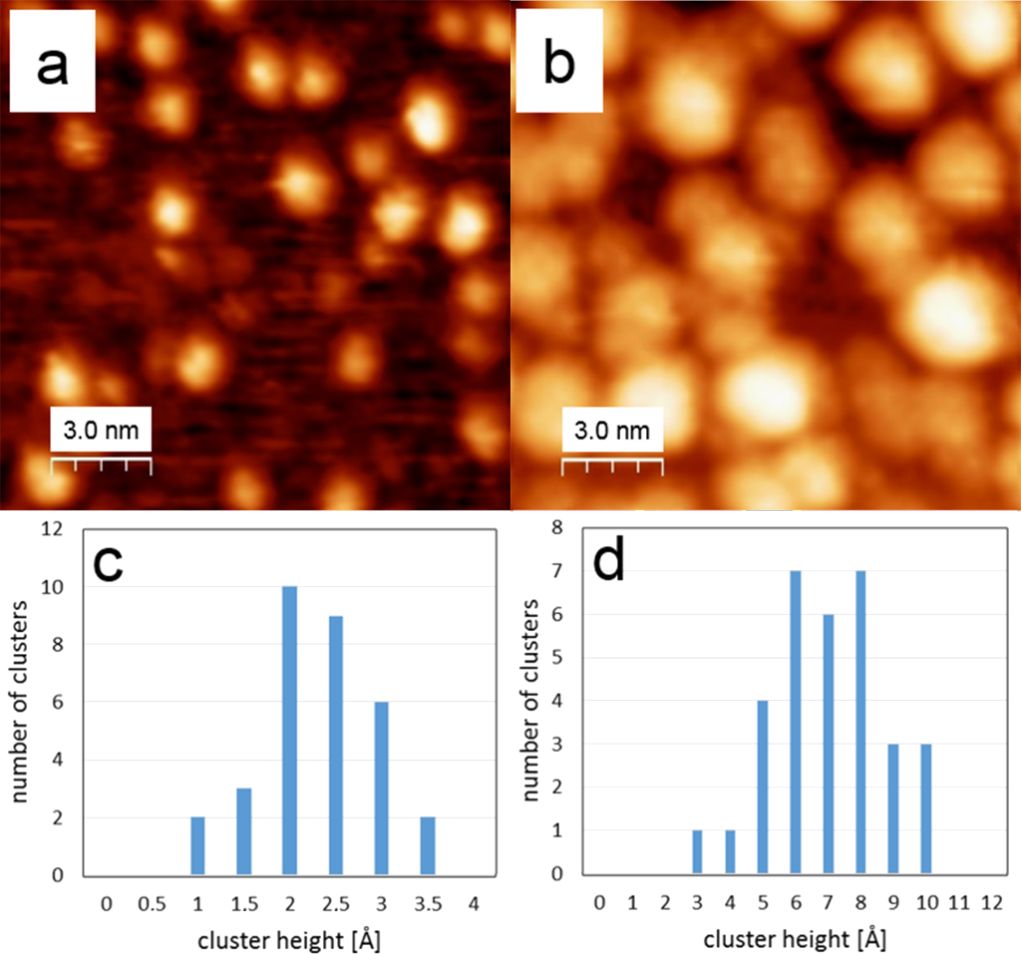
Enlarged STM images of Pd clusters on the w'-TiO*_x_* phase: (a) with the low coverage, indicating a height of one layer (2–3 Å), (b) with the high coverage, indicating a height of up to four layers (9–10 Å), (c) and (d) show the cluster height distributions from (a) and (b), respectively.

The clusters are imaged as bright spots with an almost circular shape on the oxide surface (see Figure 4a,b). For the lower coverage (Figure 3a and Figure 4a), the clusters appear to be distributed randomly on the surface and have an apparent height between 1.5 and 3 Å, as indicated by the height distribution in Figure 4c. This height corresponds to a single layer of Pd. The imaged mean diameter of a cluster is 1.9 ± 0.7 nm, as shown in the histogram in Figure 3c. At the higher coverage (Figure 3b and Figure 4b), the apparent height of the clusters increased to about 6–8 Å. This value suggests that the clusters are now 1 to 4 Pd layers thick and thus indicates that 3D growth has started. The imaged mean diameter increased to 5.5 nm with a broader size distribution of ±2.9 nm (Figure 3d).

**Figure 5:**
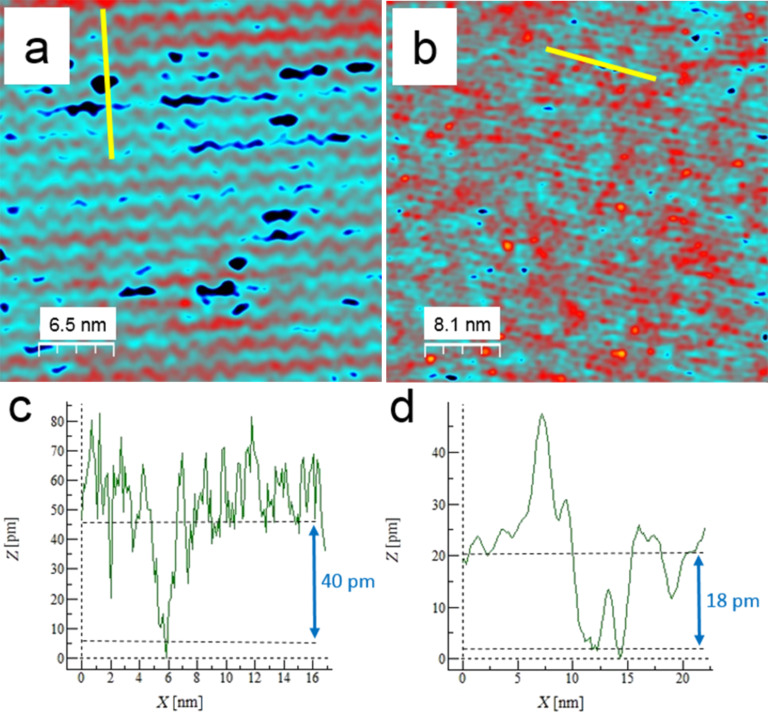
STM images of the oxide films: (a) z'-TiO*_x_* phase (32.6 × 32.6 nm^2^, bias voltage *U*_B_ = 0.97 V, *I*_T_ = 50 pA, (b) w'-TiO*_x_* phase (57.1 × 57.1 nm^2^, *U*_B_ = −1.08 V, *I*_T_ = 55 pA), (c) and (d) corresponding line profiles along the markings in image (a) and (b), respectively. Image (b) has been adapted with permission from [6], copyright 2014 American Chemical Society.

From the energetic point of view, a precondition for 3D growth (i.e., nonwetting), is that the adhesion energy of the Pd metal on the oxide is smaller than twice the surface free energy of the vacuum–metal interface of the liquid metal [19]. In the case of late transition metals on oxides, this criterion is usually met, and thus 3D growth is observed in most cases [14]. From a kinetic point of view, the growth process can be divided into two regimes: In the case of homogeneous nucleation, deposited metal atoms diffuse across a low corrugation surface and randomly combine to form stable nuclei at random positions on the surface until a saturation density is reached; then these nuclei grow in size during the further deposition process. Heterogeneous nucleation occurs on a surface of higher corrugation on which the diffusing atoms are trapped at the sites of highest adsorption energy, which thereby become preferred nucleation and growth sites. Obviously, defect sites are such traps. As shown in Figure 5a, defects in the z'-TiO*_x_* phase (depth ≈40 pm) are largely found within the trenches as an inherent property of this phase. As a consequence, the z'-TiO*_x_* phase exerts a “template effect”: The trenches are the defect locations, which in turn are the preferred nucleation sites for the Pd clusters. In turn, as shown in Figure 5b, the w'-TiO*_x_* phase contains much fewer and shallower (depth ≈18 pm) defects of no particular distribution. Therefore, under the given experimental conditions, it does not exert a noticeable “template effect”.

This result is quite in contrast to Pd cluster growth on Al_2_O_3_/Ni_3_Al(111) surfaces, where actually two different hexagonal superstructures with lattice constants of 2.4 nm and 4.1 nm act as templates for the growth of ordered metal cluster arrays [14]. A hexagonal arrangement of more equally sized clusters on the w'-TiO*_x_* Moiré structure, which is even more ideal than described in this work, is certainly achievable. This could be accomplished by varying the impact rate of Pd atoms and their mobility on the surface (i.e., the deposition rate and the substrate temperature), as was the case for Pd cluster growth on the Al_2_O_3_/Ni_3_Al(111) surface.

## Summary

Pd clusters were grown on two different, ultrathin, TiO*_x_* films, the so-called rectangular z'-TiO*_x_* and the hexagonal w'-TiO*_x_* phase. The first one consists of stripes separated by trenches and acts as a unidirectional template. This is due to the defect sites, which act as heterogeneous nucleation sites for the Pd clusters. These are an inherent property of the z'-TiO*_x_* phase and are aligned within the trenches of this structure. Along the trenches, no long-range order of the defects, and hence of the clusters, is discernible.

The w'-TiO*_x_* phase possesses a long-range (7 × 7)R21.8° superstructure, which is formed as a Moiré pattern from the superposition of the hexagonal lattices of the substrate and the oxide film. Since this structure exhibits much fewer (and shallower) defects, no “template effects” could be observed for this surface and this type of metallic cluster under the given experimental conditions.

The next reasonable steps would be (a) to attempt to achieve a hexagonal cluster distribution on the w'-TiO*_x_* phase by varying the metal deposition rate and/or the substrate temperature as was the case for metal clusters on Al_2_O_3_/Ni_3_Al(111), and (b) the investigation of the electronic as well as the adsorption and reactivity properties of the Pd clusters. In this regard, the influence of the two different titania films on these properties of clusters of the same metal is of particular interest [21].
